# Supplementary figures and images for: Sympatric versus allopatric evolutionary contexts shape differential immune response in Biomphalaria / Schistosoma interaction
Source: PLoS Pathog. 2019 Mar 20;15(3):e1007647. doi: 10.1371/journal.ppat.1007647 (PMC6443186; doi:10.1371/journal.ppat.1007647)

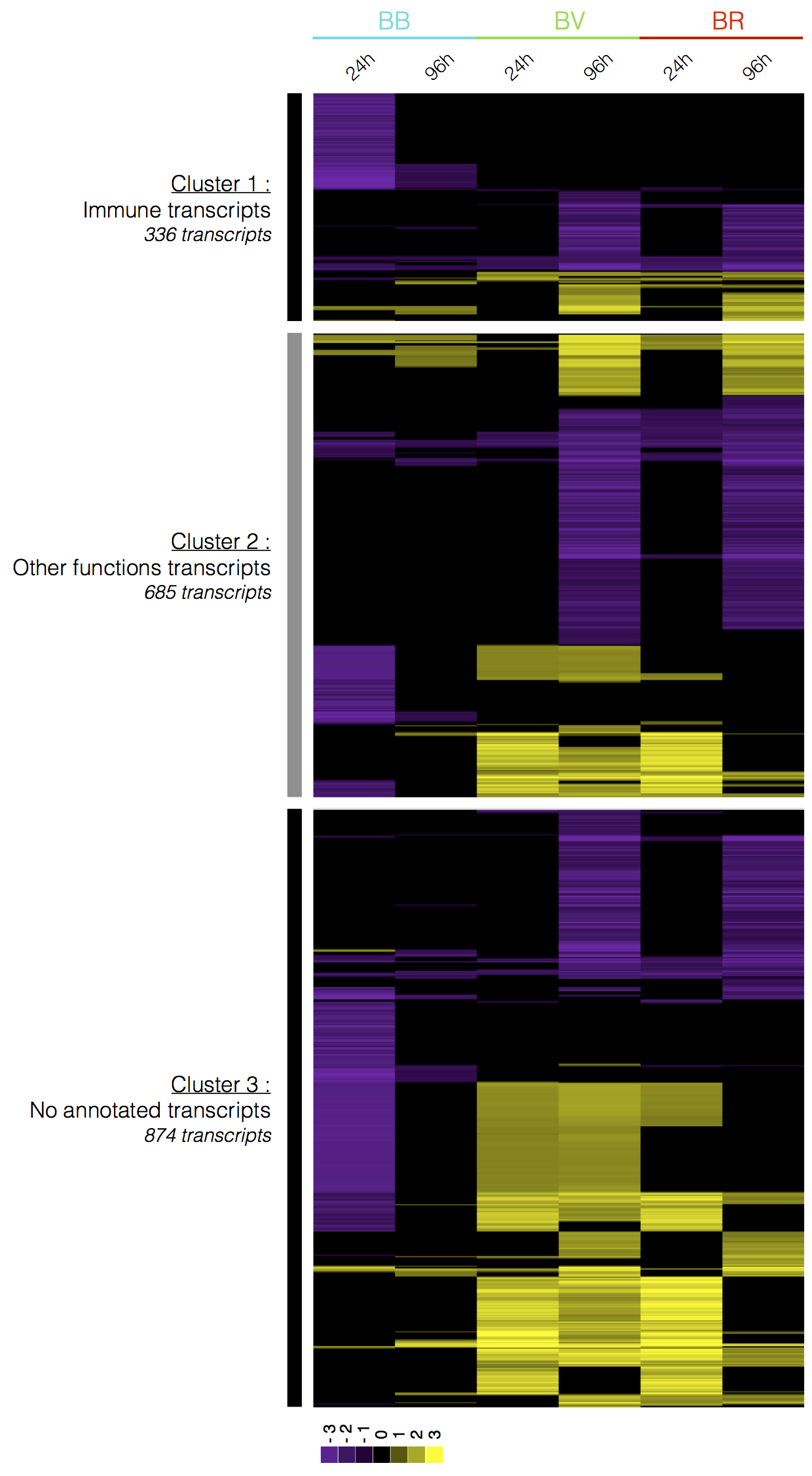

Supplement: S1 Fig — Clustering of differentially represented transcripts. Heatmap representing the profiles of the 1,895 differentially represented immune-related transcripts in the BB, BV, or BR interactions along the time course of infection (at 24 and 96 h). Each transcript is represented once and each line represents one transcript. Colors: yellow, over-represented transcripts; purple, under-represented transcripts; and black, unchanged relative to levels in control naïve snails. (TIF) [file ppat.1007647.s001.tif]

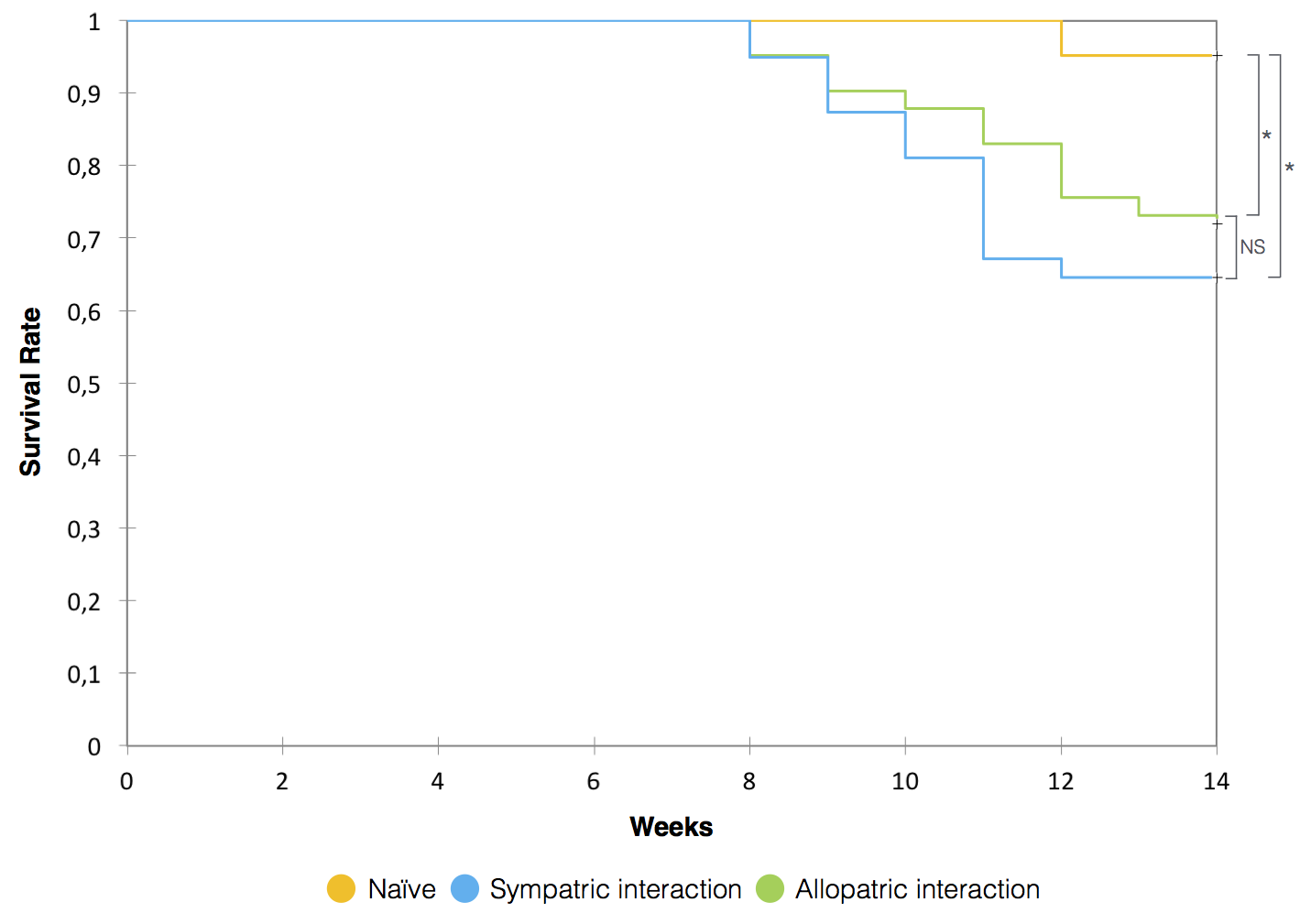

Supplement: S2 Fig — The survival rates of B. glabrata subjected to infection by different S. mansoni strains were observed over 14 weeks. Kaplan Meier graphs were generated using xlstat, and the log-rank test (p < 0.05) was used to test for significant between-group differences. Colored curves indicate the mortality rates of naïve snails (yellow) (n = 60), snails infected by the sympatric parasite (BB, BgBRE/SmBRE, blue) (n = 50), and snails infected by the allopatric parasite (BV, BgBRE/SmVEN, green) (n = 50). The difference in mortality between naïve and infected snails was significant (p<0.05), whereas that between the two infected conditions was not (p = 0.243). (TIF) [file ppat.1007647.s002.tif]

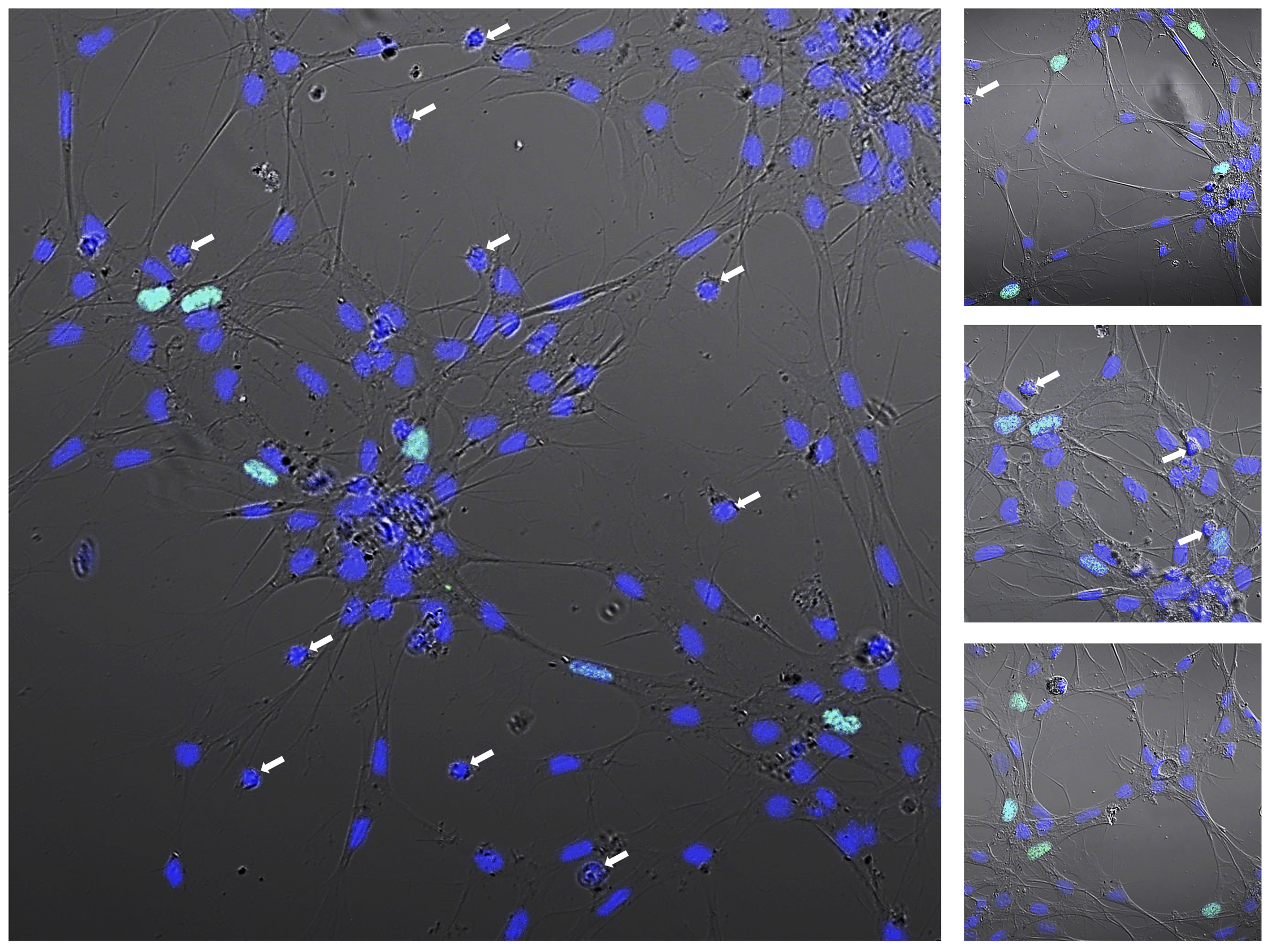

Supplement: S3 Fig — In vitro EdU labeling of hemocytes collected for in vitro analysis. Confocal microscopy of EdU-labeled hemocytes from snails subjected to the allopatric interaction BgBRE/SmVEN at 24 h post-infection (BV24). Pictures corresponded to the merge of DAPI labelling (blue); EdU labelling (green) and phase contrast pictures. The white arrows indicate the Blast-like cells. Blast-like cells were never labelled by EdU, indicating that these cells are not proliferative when circulating in the hemolymphe. Three individual snails were used for each condition. Green label: EdU-positive cells; and blue label: DAPI. Magnification x63. (TIF) [file ppat.1007647.s003.tif]

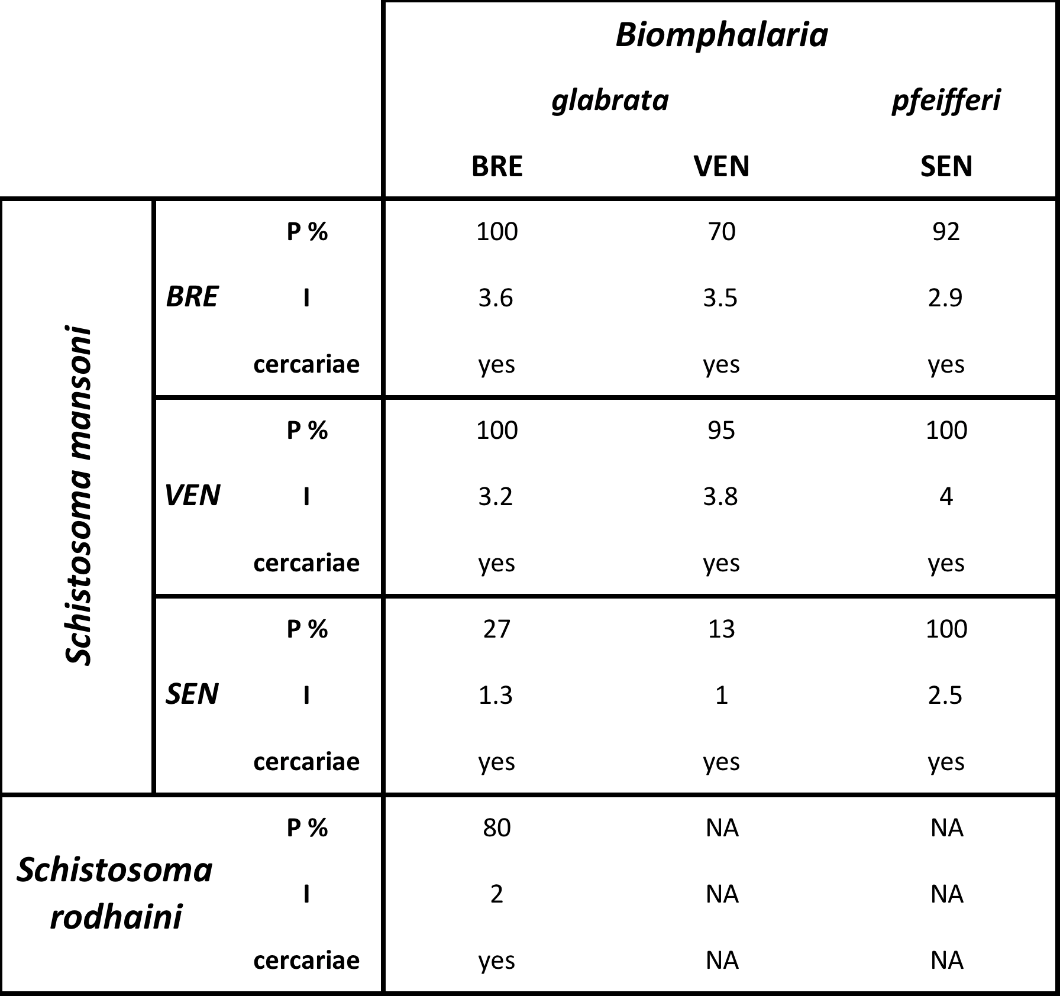

Supplement: S1 Table — The prevalence (P %: percentage of snail infected) and intensity (I: number of parasites per infected host) of infection are presented for each experimental infection. The indicated values correspond to 10 miracidia. Each pairwise combination of Biomphalaria glabrata (BgBRE, BgVEN), Biomphalaria pfeifferi from Senegal (BpSEN) and Schistosoma mansoni (SmBRE, SmVEN, SmSEN) or Schistosoma rodhaini (Srod) were tested for compatibility. The observation of cercariae shedding is also indicated. Cercariae shedding have been observed between 35 and 38 days after miracidial infections NA: non-available data. (DOCX) [file ppat.1007647.s004.docx]
